# Pediatric Pancreatic Pseudocyst Presenting as a Solid Perigastric Mass: An Uncommon Diagnostic Pitfall

**DOI:** 10.1155/crpe/6364914

**Published:** 2026-06-14

**Authors:** Giulia Maisano, Antonio Ieni, Salvatore Arena, Valeria Zuccalà, Carmelo Sofia, Pietro Impellizzeri, Carmelo Romeo, Fabiola Cassaro

**Affiliations:** ^1^ Department of Human Pathology of Adult and Childhood “Gaetano Barresi”, University of Messina, Messina, Italy, unime.it; ^2^ Biomedical and Dental Sciences and Morpho-functional Imaging, University of Messina, Messina, Italy, unime.it

**Keywords:** children, laparoscopy, pancreatic pseudocyst, pancreatitis, pediatric pancreatology, pediatric surgery

## Abstract

Pancreatic pseudocysts are rare in children and typically arise after episodes of pancreatitis or abdominal trauma. Their diagnosis generally relies on imaging studies, where they are expected to appear as fluid‐filled collections. We describe a case of a 3‐year‐old girl who has no previous occurrences of pancreatitis or trauma, in whom an unexpected heterogeneous, hypoechoic perigastric mass was incidentally detected during an ultrasound examination. The lesion demonstrated a consistent, firm appearance during an 8‐month follow‐up period and continued to be without symptoms. Laboratory examinations, including pancreatic enzymes assay, returned normal results, and imaging studies could not determine its characteristics. A laparoscopic excision was conducted for both diagnostic and treatment reasons. Histological and immunohistochemical analysis confirmed the diagnosis of a pancreatic pseudocyst. Postoperative recovery was uneventful. This case demonstrates a rare solid appearance of a pancreatic pseudocyst in a child who does not have previous pancreatic disease, emphasizing the critical importance of histopathological examination and surgical intervention when imaging results are unclear.

## 1. Introduction

Pancreatic pseudocyst in the pediatric population is a rare complication of acute pancreatitis or pancreatic trauma and is defined as a well‐circumscribed fluid collection lacking an epithelial lining, which develops as a result of inflammation and leakage of pancreatic enzymes following pancreatic injury [1, 2]. From a pathogenetic standpoint, two main types of pancreatic pseudocysts are recognized: acute pseudocysts, which typically form after acute pancreatitis or pancreatic injury and have an irregular wall; and chronic pseudocysts, which occur as a result of chronic pancreatitis and possess a well‐defined, regular, and round wall and seldom conclude without assistance [3]. The most frequently reported complications include hemorrhage, infection, and rupture.

Pancreatic pseudocysts usually present as fluid‐filled cystic lesions. In contrast, predominantly solid lesions in the absence of a clear history of pancreatitis or trauma are exceedingly rare and may be misinterpreted as mesenchymal tumors or enteric duplication cysts [4]. In this report, we present a pediatric case involving a pancreatic pseudocyst that exhibited solid characteristics, which was identified solely following surgical removal. This case highlights the crucial role of histopathological examination in the accurate diagnosis of uncommon presentations of pancreatic pseudocysts and underscores the importance of including this entity in the differential diagnosis of solid pancreatic masses in children.

## 2. Case Report

We describe the case of a 3‐year‐old girl who presented to the emergency department with persistent high fever and abdominal pain. Her parents reported a 2‐day history of fever, with temperatures reaching 39°C, associated with abdominal pain. Bowel habits were normal, and no nausea or vomiting was reported. The symptoms did not improve despite treatment with paracetamol and ibuprofen. On physical examination, the child appeared in good general condition and well hydrated; mild pharyngeal hyperemia was noted.

Initial laboratory investigations, including serum amylase and lipase levels, white blood cell count, and procalcitonin, were within normal ranges. Abdominal ultrasonography was performed to exclude intra‐abdominal pathology and incidentally revealed a rounded perigastric mass measuring approximately 21 mm in diameter. The lesion appeared heterogeneous and hypoechoic, with a detectable vascular signal, while no other abnormalities were identified. The patient was subsequently discharged with a diagnosis of acute infectious pharyngitis and, following consultation with pediatric surgeons, was enrolled in a radiological follow‐up program for further evaluation of the incidental finding.

**FIGURE 1:**
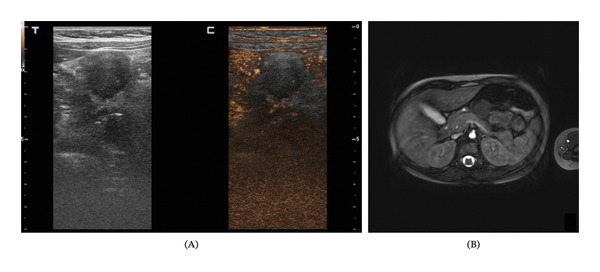
(A) 21‐mm, well‐defined, rounded hypoechoic lesion with heterogeneous internal echotexture is visualized within the perigastric fat on ultrasound. (B) MRN imaging. In the epigastric adipose tissue, at the interface between the stomach, pancreas, and proximal jejunal loops, a nodular lesion measuring approximately 22 × 23 × 24 mm is identified. The lesion appears distinctly hypointense on T2‐weighted sequences.

Three months later, a second US was performed, demonstrating stability of the lesion in terms of size and imaging characteristics (Figure 1A). The mass was located between the stomach and the pancreas and appeared partially adherent to the pancreatic parenchyma. Given the persistence of the lesion and the uncertainty regarding its nature, contrast‐enhanced magnetic resonance imaging was performed. After a further 3 months of follow‐up, US followed by contrast‐enhanced MRI was performed. MRI confirmed the location and size of the mass (22 × 23 × 24 mm) and showed no evidence of internal vascularization. The lesion exhibited predominantly fibrotic and myofibrotic features with low cellularity (Figure 1B), findings that did not allow a definitive preoperative diagnosis.

**FIGURE 2:**
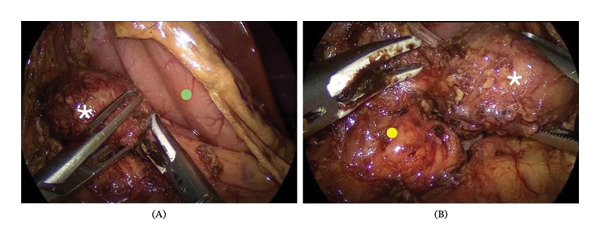
Intraoperative images. (A) The lesion (white asterisk) was exposed through the created window in the epiploic retro‐cavity, adjacent to the greater curvature of the stomach (green dot). (B) Circumferential dissection of the lesion (white asterisk) revealed posterior continuity with the pancreatic body (yellow dot). Complete mobilization and isolation of the mass were achieved using a vessel‐sealing device.

After multidisciplinary discussion involving pediatric surgeons and radiologists, surgical exploration was considered appropriate to establish a definitive diagnosis, given the inconclusive imaging findings and the proximity of the lesion to the pancreas. Two months later, a final preoperative US was performed prior to surgery, showing no changes in size or appearance. A laparoscopic approach was selected to allow both diagnostic and therapeutic management. Through laparoscopic access to the omental bursa, a well‐circumscribed mass was identified near the greater curvature of the stomach (Figure 2A). Careful dissection revealed continuity with the body of the pancreas. The lesion was completely mobilized and excised using a vessel‐sealing device, with meticulous hemostasis maintained throughout the procedure (Figure 2B). The specimen was retrieved in an endoscopic bag through the umbilical incision, and an abdominal drain was placed after confirmation of the absence of pancreatic or biliary leakage.

The postoperative course was uneventful. Oral feeding was resumed on the second postoperative day, and the abdominal drain was removed the same day. The patient remained asymptomatic, and surgical wounds healed without complications. She was discharged home in good clinical condition on the fifth postoperative day. No complications were recorded during the 6‐month follow‐up.

**FIGURE 3:**
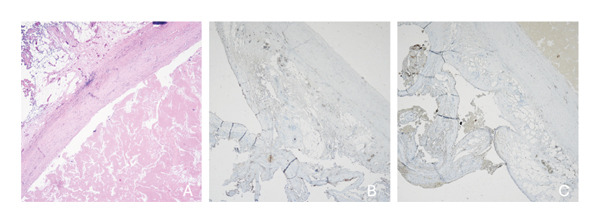
H&I staining. (A) Hematoxylin‐eosin 4X. Histological section of the cyst wall. A cystic cavity is lined by a fibrous wall showing mild chronic inflammatory infiltrate, predominantly lymphocytic, and no evident epithelial lining. The lumen contains eosinophilic amorphous material. (B) Immunohistochemistry for calretinin (4X). No significant staining is observed along the cyst wall, ruling out a mesothelial origin. (C) Immunohistochemistry for cytokeratins (CK, 4X). The staining is essentially negative, indicating the absence or marked attenuation of residual epithelial cells along the cyst wall.

Histopathological examination revealed a lesion characterized by a cystic wall composed of dense fibrous tissue, enclosing mild chronic inflammatory infiltrate, dystrophic calcifications, and cholesterol crystals. No epithelial lining was identified, a key feature supporting the diagnosis of a pancreatic pseudocyst rather than a true cystic lesion. Immunohistochemical staining for cytokeratin AE1/AE3, D2‐40, and calretinin was negative, confirming the absence of epithelial or mesothelial lining (Figure 3A, B, C). On the basis of these findings, a final diagnosis of pancreatic pseudocyst was established.

## 3. Discussion

A pancreatic pseudocyst is a fluid collection rich in pancreatic secretions and debris, surrounded by a fibrous wall without an epithelial lining. It typically arises as a complication of pancreatitis. While pseudocysts represent 10%–15% of pancreatic cystic lesions in adults, they are uncommon in children, occurring in only a small proportion of pediatric pancreatitis cases [5]. Pancreatic pseudocysts are generally considered to arise because of an underlying acute or chronic pancreatic insult [6]. The pathogenesis is primarily attributed to disruption of the pancreatic ductal system, leading to extravasation of enzyme‐rich pancreatic secretions into the surrounding peripancreatic tissues. This inflammatory fluid collection is subsequently walled off by a nonepithelialized fibrous capsule [7]. Pancreatic pseudocysts account for most pancreatic cystic lesions. Their reported incidence ranges from approximately 10% to 20% following an episode of acute pancreatitis and 20% to 40% in the setting of chronic pancreatitis [8].

In pediatric patients, suspicion of pancreatic pseudocysts arises primarily following trauma or pancreatitis. However, a few rare cases not associated with either trauma or pancreatitis have been reported in the literature [9, 10], although these remain exceptional. In our clinical case, however, neither the clinical history nor laboratory findings suggested trauma or pancreatitis. Moreover, the child’s presentation differed from what is typically described in the literature, making the initial diagnostic suspicion of a pancreatic pseudocyst particularly challenging.

Imaging studies are essential for confirming the diagnosis of pancreatic pseudocysts, with ultrasound, CT, and MRI being the most common modalities [11]. MRI provides excellent intrinsic tissue characterization, but in children (under 4–5 years of age), it often requires general anesthesia [11]. In our case, radiological findings alone were insufficient to establish a definitive diagnosis. The patient was managed with a conservative follow‐up approach, in agreement with the radiologist and pediatric oncologist, due to the absence of symptoms, the stability of the lesion, and its nonaggressive behavior over time; histological examination following surgical excision ultimately confirmed the nature of the lesion. Ultrasound was useful for identifying the lesion’s position and morphology and allowed serial noninvasive and radiation‐free follow‐up. Subsequent MRI with contrast allowed for detailed tissue characterization and excluded vascular involvement, confirming the mass as fibrotic and hypovascular. MRI provides soft‐tissue characterization without the radiation exposure associated with CT scans [11].

The need for treating pancreatic pseudocysts in pediatric patients remains debated. Pseudocysts typically mature over 2–6 weeks; approximately one‐third resolve spontaneously, while the remainder may require intervention to prevent complications such as infection, hemorrhage, or rupture [12].

Treatment of pancreatic pseudocysts is generally indicated only in symptomatic or complicated cases, such as those with persistent pain, infection, progressive enlargement, biliary obstruction, or compression of adjacent organs. Therapeutic options include percutaneous drainage, endoscopic drainage, and surgery [13]. Minimally invasive approaches are preferred when feasible: Percutaneous drainage achieves high success rates (around 90%), and endoscopic drainage is particularly effective when the pseudocyst is adjacent to the stomach or duodenum, with outcomes comparable to surgery but with lower morbidity [14].

Surgery is therefore reserved for selected cases, such as when drainage is not feasible or when the etiology and nature of the lesion remain uncertain. Etiology may also influence management: Pseudocysts following trauma tend to resolve more frequently without intervention, possibly due to better parenchymal healing, whereas those unrelated to trauma are more likely to require treatment. Current recommendations emphasize that intervention should be based on symptoms, complications, and ductal involvement rather than size alone [15]. Such treatment considerations apply only when a definitive diagnosis of pancreatic pseudocyst has been established.

In our case, the diagnosis was confirmed only after histological examination. The patient presented with an asymptomatic heterogeneous, hypoechoic perigastric mass adherent to the pancreas, with stable imaging over several months and without clinical or laboratory evidence of pancreatitis.

Pancreatic pseudocysts in the pediatric population most commonly arise as a consequence of pancreatic injury, particularly abdominal trauma or pancreatitis. Recent evidence confirms that trauma represents one of the leading causes in children [1, 10]. A differential diagnosis included lymphangioma, enteric duplication cyst, and mesenchymal lesions [10]. More generally, pancreatic cystic lesions can be classified into nonneoplastic lesions, such as pseudocysts, and neoplastic cysts, including intraductal papillary mucinous neoplasms, mucinous cystic neoplasms, serous cystadenomas, solid pseudopapillary neoplasms, and cystic neuroendocrine tumors [16, 17]. Additionally, other cystic lesions should be considered. Splenic, mesenteric, and omental cysts are anatomically separate from the pancreas and lack pancreatic enzymes [18], while rare choledochal cysts may mimic pseudocysts [19]. Diagnosis relies on clinical, imaging, and histological correlation.

Because the lesion persisted and its nature remained unclear, laparoscopic excision was performed. Although conservative management is suitable for confirmed, asymptomatic pseudocysts, surgical removal is recommended when the diagnosis is uncertain.

These atypical features demonstrate the limitations of imaging when pseudocysts lack fluid components or symptomatic correlation. In asymptomatic cases with unclear radiologic features, surgical removal may remain the only reliable diagnostic and therapeutic option. Although conservative management is preferred for confirmed pseudocysts, an uncertain diagnosis warrants excision to exclude malignancy or other noninflammatory cystic lesions.

This case shows that a pancreatic pseudocyst can present unusually, without fluid content and in the absence of prior pancreatitis or abdominal trauma. When clinical findings are inconclusive and imaging does not allow a clear characterization of the lesion, surgical resection with subsequent histopathological analysis remains essential to establish a definitive diagnosis and to exclude potential malignancy.

## Funding

The authors received no funding.

Open‐access publishing was facilitated by Universita degli Studi di Messina, as part of the Wiley–CRUI‐CARE agreement.

## Consent

Written informed consent for participation and publication was obtained from the patient’s parents.

## Conflicts of Interest

The authors declare no conflicts of interest.

## Data Availability

The data that support the findings of this study are available on request from the corresponding author. The data are not publicly available due to privacy or ethical restrictions.
